# On the role of immunity and genetic interactions in Alzheimer’s Disease: The interplay between COMT and MIR146a is significantly associated with AD

**DOI:** 10.1002/alz70861_108665

**Published:** 2025-12-23

**Authors:** Anatoliy I Yashin, Deqing Wu, Konstantin Arbeev, Olivia Bagley, Hongzhe Duan, Rachel Holmes, Svetlana Ukraintseva

**Affiliations:** ^1^ Social Science Research Institute, Duke University, Durham, NC USA; ^2^ Duke University, Durham, NC USA

## Abstract

**Background:**

Accumulating evidence points to a major role of compromised immunity in Alzheimer’s Disease (AD). The MIR146a and COMT genes are both known for their involvement in immune responses, as well as in AD‐related traits. COMT also regulates the metabolism of catecholamines, which is linked to inflammation and trained immunity. We hypothesized that the interplay between MIR146a and COMT genes may influence AD risk.

**Method:**

To test this hypothesis, the logistic regression model with the interaction term was applied to the analysis of the Cardiovascular Health Study (CHS) data, males and females combined. A binary AD trait was created: bn_AD_2 =1 (case) included participants who survived age 85 and did not have AD before or after this age (*N* =1,421); bn_AD_2 =0 (control) included participants who acquired AD at any age (*N* =257). Framingham Heart Study (FHS) data were used for replication. The case and control groups there included 903 and 354 participants, respectively.

**Result:**

The analysis of CHS data found that the interaction between SNPs rs2910163 in *MIR146a* and rs4818 in *COMT* genes is significantly associated with AD occurrence (β=‐0.48, *p* =1.62E‐02, Bonferroni=1.67E‐02). This finding was confirmed in FHS data, for the same SNPs (β=‐0.41, *p* =3.63E‐02). Notably, rs2910163 is in strong LD with rs2910164, and both rs2910164 and rs4818 have been associated with multiple health‐related traits in other studies.

**Conclusion:**

Our study findings emphasize the importance of factors regulating immune responses in AD development and support the role of GxG interactions in AD risk.

